# S1PR4-dependent effects of Etrasimod on primary human myeloid immune cell activation

**DOI:** 10.3389/fphar.2025.1590816

**Published:** 2025-04-24

**Authors:** Fiona K. Sailer, Megan A. Palmer, Blerina Aliraj, Jan Heering, Andreas Brockmann, Mohammed A. F. Elewa, Aissa Röhrig, Ewgenij Proschak, Dariusz T. Stepniak, Simeon Ramsey, Bernhard Brüne, Andreas Weigert

**Affiliations:** ^1^ Institute of Biochemistry I, Faculty of Medicine, Goethe University Frankfurt, Frankfurt am Main, Germany; ^2^ Fraunhofer Institute for Translational Medicine and Pharmacology ITMP, Frankfurt am Main, Germany; ^3^ Fraunhofer Cluster of Excellence Immune-Mediated Diseases CIMD, Frankfurt am Main, Germany; ^4^ Biochemistry Department, Faculty of Pharmacy, Kafrelsheikh University, Kafr El-Sheikh, Egypt; ^5^ Department of Internal Medicine 1, Goethe University Hospital, Frankfurt am Main, Germany; ^6^ Institute of Pharmaceutical Chemistry, Goethe University Frankfurt, Frankfurt am Main, Germany; ^7^ Arena Pharmaceuticals, Pfizer Inc., New York, NY, United States; ^8^ Inflammation and Immunology, Pfizer Inc., Cambridge, MA, United States; ^9^ Frankfurt Cancer Institute, Goethe University Frankfurt, Frankfurt am Main, Germany

**Keywords:** Sphingosine-1-phosphate, S1P receptor modulator, S1PR4, Etrasimod, inflammatory bowel disease

## Abstract

**Background:**

Sphingosine-1-phosphate (S1P) and its five receptors S1PR1-5 play an essential role in the migration, differentiation and activation of various immune cells. Several S1PR modulators with distinct selectivity have been recently approved for the treatment of various inflammatory diseases. Among those are Ozanimod, an S1PR1/5 modulator approved for the treatment of ulcerative colitis and multiple sclerosis, and Etrasimod, an S1PR1/4/5 modulator approved for the treatment of ulcerative colitis. Chronic autoinflammatory diseases such as the inflammatory bowel diseases (IBDs) Crohn’s disease and ulcerative colitis are characterized by an abundance of disease-propagating immune cells in the gastrointestinal tract. Since currently available treatment options such as biologics provide a sometimes inadequate treatment response, one alternative strategy to treat IBDs is the use of S1P receptor modulators.

**Objective:**

We aimed to investigate if targeting S1PR4 affects the impact of Etrasimod on the activation of primary human immune cells, and to elucidate the mode of action of Etrasimod on S1PR4.

**Methods:**

Primary human macrophages, plasmacytoid dendritic cells and neutrophils were pretreated with S1P, Etrasimod (S1PR1/4/5), Ozanimod (S1PR1/5), Siponimod (S1PR1/5), CYM 50308 (S1PR4 agonist) and CYM 50358 (S1PR4 antagonist), and then stimulated with Zymosan A, ODN 2336 and PMA, respectively. We measured cytokine and chemokine production by macrophages and plasmacytoid dendritic cells via CBA/Legendplex, and survival and activation markers for neutrophils via flow cytometry. Confocal microscopy of S1PR-expressing CHO-K1 cell lines was used to study receptor internalization.

**Results:**

We found that signaling induced by S1P, Etrasimod and the S1PR4 agonist attenuates CCL20 and CXCL5 production by Zymosan-stimulated macrophages, and these findings were confirmed by S1PR4 knockdown. Additionally, S1PR4 was involved in the regulation of IFN-α production by ODN2336-stimulated plasmacytoid dendritic cells. Lastly, both Etrasimod and the S1PR4 agonist reduced the activation level of PMA-stimulated neutrophils. Regarding receptor dynamics, we show that Etrasimod induces internalization of S1PR4.

**Conclusion:**

Taken together, our data show that S1PR4 takes on an essential role in the regulation of various immunological functions, and that Etrasimod can act as a superagonist/functional antagonist of S1PR4.

## 1 Introduction

Sphingosine-1-phosphate (S1P) is a sphingosine-derived lipid mediator that binds to specific GPCRs (S1PRs), with five receptor subtypes being described that are expressed on several cell types and in numerous tissues. Their expression levels differ across those cell types, and they are coupled to different G proteins that induce various downstream signaling events (Verstockt et al., 2022). Based on its broad distribution, S1P signaling in general is involved in a multitude of physiological processes such as cardiac development, vascular homeostasis, immune cell function and neurological regulation (Cartier and Hla, 2019). More specifically, S1PR1, for instance, is ubiquitously expressed by immune cells and the vasculature. It is essential in regulating lymphocyte migration along a S1P concentration gradient between the blood, lymph and extra-vascular tissues. S1PR1, as well as S1PR2 and S1PR3, are generally present in most tissues. S1PR4, however, is more exclusively expressed in the hematopoietic and lymphoid, and S1PR5 in the central nervous system (Verstockt et al., 2022). As for the coupled G proteins and subsequent signaling pathways, S1PR1, S1PR4 and S1PR5 all activate Gi, while S1PR4 and S1PR5 couple additionally to G12/13. Moreover, all S1PR are capable of G-protein independent signaling via the β-arrestin pathway, which also leads to receptor internalization (Gaidarov et al., 2024).

The involvement of S1P signaling in regulating immunological responses makes it a promising target for the treatment of various inflammatory diseases such as multiple sclerosis and the inflammatory bowel diseases (IBDs) Crohn’s disease (CD) and ulcerative colitis (UC). While IBDs differ in their clinical presentation–UC is limited to the colon and characterized by superficial mucosal inflammation that can end in ulcerations, while CD can affect the whole digestive tract and is characterized by transmural inflammation that can lead to abscesses–they both share some of the underlying disease mechanisms (Kobayashi et al., 2020; Roda et al., 2020). At the cellular level, macrophages produce an overabundance of pro-inflammatory cytokines such as tumor necrosis factor (TNF)-α, interleukin (IL)-6, IL-12 and IL-23 upon bacteria uptake. Neutrophilic granule release as well as neutrophil extracellular trap formation are elevated. CD4^+^ T cells differentiate into effector Th1 and Th17 subsets, which accumulate in the intestinal tissue and produce pro-inflammatory cytokines such as IFN-γ and IL-17. Additionally, chemokines such as CCL20 are essential in redirecting pro-inflammatory immune cell subsets such as Th17 cells during intestinal inflammation (Chang, 2020; Kobayashi et al., 2020; Roda et al., 2020; Verstockt et al., 2022). The etiology of IBDs is complex and not yet fully understood. An elaborate synergy between genetic, immunological, microbial and environmental factors is the likely root cause of the rising global incidence of both CD and UC (Sabino et al., 2019). While there are various treatment options available for IBDs depending on disease severity, response to those is sometimes lacking, and adverse effects abound (Kotla and Rochev, 2023; Sabino et al., 2019). Hence, S1PR modulators offer a promising alternative treatment strategy. Since S1PR1 is crucial for the egress of lymphocytes from the lymph nodes, S1PR1 agonists can block this process by inducing the internalization and subsequent degradation of the receptor, and hence reducing the degree of inflammation in the affected tissues. Fingolimod, a S1PR1,3-5 modulator was the first of its class to be discovered. While it was approved for the treatment of multiple sclerosis, it showed some adverse effects such as bradycardia. However, more recently developed S1PR modulators that are more selective were shown to be advantageous in having fewer side-effects, while still providing the desired immune-paralytic effect. Two such newer S1PR modulators are Ozanimod (S1PR1/5) and Etrasimod (S1PR1/4/5) (Grossberg et al., 2022). Ozanimod was approved for treatment of moderate-severe UC in 2021 after achieving significant amelioration in clinical remission, endoscopic improvement and mucosal healing (Fudman et al., 2024). Etrasimod was approved for treatment of moderate-severe UC in 2023, after achieving similar success regarding the endpoints mentioned for Ozanimod. Etrasimod also has a favorable safety profile compared to other S1P modulators, showing no increased risk of infection and similar risk profiles in regard to bradycardia and macular oedema. Additionally, the half-life and wash-out period of Etrasimod is comparably short, which allows for fast lymphocyte recovery after end of treatment (Fudman et al., 2024; Sandborn et al., 2023). Overall, Etrasimod has been shown to have a favorable pharmacological profile in the treatment of moderate-severe UC compared to other S1PR modulators. Since Etrasimod stands out among other similar drugs by being able to activate S1PR4, the question emerges whether this activity has any impact on the function of the immune system.

While the immune-modulatory properties of S1PR4 have not yet been fully elucidated, its expression pattern and some aspects of its role in regulating immune cell function are well documented. For instance, S1PR4 knockout in mice has been shown to decrease the percentage of T_H_17 cells as well as cytokine production by dendritic cells (Schulze et al., 2011). The maturation of plasmacytoid dendritic cells (pDCs) has been found to be impeded by S1PR4 knockout in mice (Dillmann et al., 2015), while for human pDCs, S1PR4 signaling has been found to attenuate IFN-α production upon TLR stimulation (Dillmann et al., 2016). Another study in S1PR4 knockout mice showed reduced chemokine production and macrophage infiltration into the inflamed tissue (Schuster et al., 2020). Furthermore, ablation of S1PR4 in mice showed impaired B cell chemotaxis (Riese et al., 2022) as well as impaired neutrophil tissue homing (Luker et al., 2024). In a slightly different perspective, S1PR4 knockout in mice has been found to be beneficial in a cancer setting since it limited tumor progression through the accumulation of tumor-suppressive CD8^+^ T lymphocytes (Olesch et al., 2020).

Taken together, there is compelling evidence of S1PR4 involvement in immune regulation, although there is still a need for more investigation, especially in the human immune system. Our study aimed to address the question which role S1PR4 specifically plays in the context of inflammatory diseases such as IBDs, and to identify the mode of action of Etrasimod at the S1PR4.

## 2 Materials and methods

### 2.1 Reagents

Sphingosine-1-Phosphate (d18:1) (1 mM; Avanti Polar Lipids) was dissolved in a buffer containing 50 mM HEPES, 100 mM NaCl, 10 mM MgCl2, 0.1% fatty-acid free BSA (pH 7.4). Ozanimod (2 mM; Cayman Chemical), Etrasimod (5 mM; Pfizer), Siponimod (5 mM; Cayman Chemical), CYM 50308 (5 mM; Cayman Chemical), CYM 50358 hydrochloride (5 mM; Tocris), phorbol 12-myristate 13-acetate (PMA; 0.01 mM; Sigma-Aldrich) and Pan Caspase OPH Inhibitor Q-VD (10 mM; R&D Systems) were dissolved in DMSO. CpG-A (2336) ODN (500 μM; InvivoGen) was dissolved according to manufacturer’s instructions. Zymosan A from *saccharomyces cerevisiae* (1 mg/ml; Sigma-Aldrich) was dissolved in RPMI 1640 medium.

### 2.2 Primary human immune cell isolation and cell culture

PBMCs were obtained from Buffy Coats (DRK-Blutspendedienst Baden-Württemberg-Hessen) using Pancoll (PAN Biotech) gradient centrifugation. PBMCs were cultured in 6-well plates in RPMI 1640 (Gibco) containing 2% FBS (Capricorn Scientific). Primary monocytes were magnetically purified from PBMCs using a CD14^+^ positive selection kit (Stemcell) according to the manufacturer’s instructions. Monocytes were differentiated into macrophages in TC-treated 6-well plates with Macrophage-SFM (1X) (Gibco) + 1% Penicillin/Streptomycin (Sigma-Aldrich) under addition of 50 ng/ml M-CSF (Immunotools) for 6 days. On day 6, medium was changed to RPMI 1640 + 10% FBS + 1% Penicillin/Streptomycin, and on day 7, the macrophages were ready for treatment. Treatment consisted of 100 nM S1PR modulator pre-incubation overnight, and further 24 h incubation after addition of 100 nM S1P and 50 μg/ml Zymosan. Primary pDCs were magnetically purified from PBMCs using a pDC purification kit (Stemcell) according to the manufacturer’s instructions. pDCs were cultured in 96-well plates in X-Vivo10 (Lonza) supplemented with 2.5% human serum, 1% Penicillin/Streptomycin and 20 ng/ml IL-3 (Immunotools). pDCs were pre-treated with 100 nM S1PR modulator for 30 min and 100 nM S1P for an additional 30 min. Then, 5 μg/ml ODN 2336 were added and cells were incubated overnight. Neutrophils were obtained from Buffy Coats via ACD/Dextran density separation with subsequent erythrocyte lysis using H_2_O and KCl. Neutrophils were cultivated in RPMI 1640 + 10% FBS + 1% Penicillin/Streptomycin, with addition of 0.1 μM Pan-Caspase-Inhibitor (PCI). After PCI incubation overnight, neutrophils were pre-treated with 100 nM S1PR modulators for 2 h. Then, 100 nM S1P and 20 nM PMA were added and neutrophils were incubated overnight. All cells were cultivated at 37°C and 5% CO_2_ during both maintenance and treatment.

### 2.3 RNA isolation and RT-qPCR

RNA was isolated using TRIzol reagent (Invitrogen) according to manufacturer’s instruction followed by cDNA transcription with the Maxima First Strand cDNA Synthesis Kit for RT-qPCR (Thermo Scientific). Real-time qPCR was performed using the QuantStudio 3 system (Applied Biosystems) and PowerUP SYBR Green Master Mix (Applied Biosystems). Primers against S1PR4 and TATA-box binding protein (TBP) were from Qiagen. Additional primers (Biomers) were S1PR1 sense 5′ tct​gct​ggc​aaa​ttc​aag​cga 3′ and antisense 5′ gtt​gtc​ccc​ttc​gtc​ttt​ctg 3′, S1PR2 sense 5′ cat​cgt​cat​cct​ctg​ttg​cg 3′ and antisense 5′ gcc​tgc​cag​tag​atc​gga​g 3′, S1PR3 sense 5′ gga​tgt​gct​ggc​tca​ttg​c 3′ and antisense 5′ cag​gat​ggt​aga​gca​gtc​agg 3′ and S1PR5 sense 5′ gcg​cac​ctg​tcc​tgt​act​c 3′ and antisense 5′ gtt​ggt​gag​cgt​gta​gat​gat​g 3´. TBP expression was used for normalization. RT-qPCR results were quantified using QuantStudio Design & Analysis Software (Applied Biosystems).

### 2.4 Cytokine quantification

IL-6 or IFN-α in cell culture supernatants were quantified using Cytometric bead array (CBA) Flex Sets (BD Biosciences), chemokines were quantified using the LEGENDplex™ HU Proinflam. Chemokine Panel 1 (Biolegend). Samples were acquired by flow cytometry and processed with FlowJo™ v10.8 Software (BD Life Sciences) and GraphPad Prism version 10.0.2 (GraphPad Software).

### 2.5 Flow cytometry

Single-cell suspensions were blocked with FcR blocking reagent (Miltenyi Biotec) in 0.5% PBS-BSA for 10 min, stained with fluorochrome-conjugated antibodies for 20 min and analyzed on a FACSSymphony A5SE flow cytometer (BD Biosciences). Live single cells were identified by FSC/SSC characteristics. Data were analyzed using FlowJo™ v10.8 Software (BD Life Sciences). All antibodies and secondary reagents were titrated to determine optimal concentrations. Comp-Beads (BD) were used for single-color compensation to create multicolor compensation matrices. For gating, fluorescence minus one controls were used. The instrument calibration was controlled daily using Cytometer Setup and Tracking beads (BD Biosciences). The following antibodies and dyes were used: anti-CD15-BUV805, anti-CD11b-PE-CF594 (both BD Biosciences), anti-CD11b(activation epitope)-APC (Invitrogen), S1PR4-AF405 (R&D Systems), AnnexinV-FITC (Immunotools), Zombie UV Fixable Viability Kit (Biolegend) and 7-AAD (BD Biosciences).

### 2.6 Macrophage transfection

For knockdown of S1PR4, macrophages were incubated in serum-free RPMI 1640 + 1% Penicillin/Streptomycin for at least 16 h. Then, ON-TARGETplus siRNA, either S1PR4 SMARTpool or Non-targeting Control Pool (50 nM; Dharmacon) was added to the cells together with HiPerFect transfection reagent (Qiagen) and serum-free medium. After 6 h, 1 ml of serum-free medium was added. The next morning, medium was changed back to serum-containing medium, and macrophages were incubated for a total of 72 h before subsequent treatment, collection of supernatants (for chemokine quantification) and RNA isolation.

### 2.7 Generation of stable S1PR1 and S1PR4 cell lines

CHO-K1 cells (DSMZ, ACC 110) were maintained at 5% CO_2_ and 37°C using Ham’s F-12 medium (Gibco) supplemented with 10% FBS and 1% penicillin/streptomycin (Sigma Aldrich). For splitting, cells were harvested every 3 to 4 days using Trypsin/EDTA. Standard tissue culture (TC) plastics were used.

A CHO-K1 cell line with stable expression of S1PR1 and one with stable expression of S1PR4 was generated using the sleeping beauty method (Kowarz et al., 2015) with the pSBbi-Bla-T8-SNAP-S1PR1 and pSBbi-Bla-T8-SNAP-S1PR4 plasmids (see Supplementary Figure S1). Stable insertion of the expression cassettes into the host genome was facilitated by the transposase expressed from the plasmid pcGlobin SB100xco (see Supplementary Figure S1), which was co-transfected using Lipofectamine 3000 (Invitrogen). After selection with Blasticidin (10 mg/ml, InvivoGen) all positive cells harbor at least one copy of the S1PR1 or S1PR4 expression cassettes, respectively, at a random position in their genome. Success of the integration was confirmed with RT-qPCR as described above with the same S1PR1 and S1PR4 primers, with Cog1 (biomers, sense 5′ act​agc​ctc​cag​cca​gat​ca 3′ and anti-sense 5′ gca​ggt​gag​tcg​tct​tcc​tc 3′) used for normalization.

### 2.8 Western blotting

Cell lysates were resolved on polyacrylamide gels followed by semi-dry transfer onto nitrocellulose membranes. Membranes were blocked with 5% milk/100 mM Tris–HCl, 150 mM sodium chloride, 0.05% (v/v) Tween 20 (TTBS) followed by incubation with Revert 700™ Total Protein Stain (LI-COR Biosciences) and Revert™ Wash Solution (LI-COR Biosciences) and with a monoclonal antibody against S1PR4 (Sigma-Aldrich). For protein detection, the membrane was incubated with IRDye secondary antibody (LI-COR Biosciences) in 5% milk/TTBS. Membranes were visualized with the Odyssey infrared imaging system (LI-COR Biosciences) and quantified with Image Studio Digits 5.0 (LI-COR Biosciences). The signal of primary antibody was normalized to the respective total protein.

### 2.9 Confocal microscopy

CHO-K1 wt, CHO_S1PR1 and CHO_S1PR4 cells were seeded in Poly-L-Lysine-coated µ-Slides 8 Well high Glass Bottom (ibidi) the day before staining and treatment. Cells were stained with SNAP-Surface 649 (New England Biolabs) according to manufacturer’s instructions, but instead of full medium, Tag-lite SNAP/CLIP Labeling Medium (Revvity) was used. Then, cells were treated with 100 nM S1P, Etrasimod or Ozanimod for 30 min or 60 min at 37°C and 5% CO_2_. Afterwards, cells were washed with PBS, fixed in 4% paraformaldehyde (ROTI®Histofix, Carl Roth) for 10 min at room temperature, and washed three times in PBS. Finally, cells were stained with 20 nM MemGlow™ 488 (Cytoskeleton, Inc.) in PBS for 10 min at room temperature, and washed three times in PBS. Cells were kept in PBS for subsequent microscopy, which was done on the same day. Confocal microscopy was performed using a Zeiss laser scanning microscope 800 Axio-Observer using Zen Blue v2.3 software for acquisition (Carl Zeiss). All images were acquired using GaAsP detectors with a Plan-Apochromat 40x/1.4 Oil DIC M27 objective. 488 nm and 640 nm diode lasers were used at 0.5% and 1.5%, respectively, and detector gain was set to 800 V. Images were acquired at 16 bit with a scaling of 0.085 μm × 0.085 μm per pixel.

### 2.10 Image analysis

Image analysis was performed using macros in Fiji (Schindelin et al., 2012). Briefly, intensity analysis for mean pixel intensity was performed on segmented images for plasma membrane and cytoplasm. Segmentation was performed with the cellpose algorithm, using the pre-trained model cyto3 as a basis for a model self-trained specifically on CHO cells (Pachitariu and Stringer, 2022; Stringer et al., 2021; Stringer and Pachitariu, 2024). Macros are available upon request.

### 2.11 Statistical analysis

Data were analyzed using GraphPad Prism 10.0.2 (GraphPad Software). p-values were calculated using either one-way ANOVA, one sample t-test or Kruskal-Wallis test as indicated in the respective Figure legends.

## 3 Results

### 3.1 Involvement of S1PR4 signaling in Zymosan-mediated IL-6 production by macrophages is limited

**FIGURE 1:**
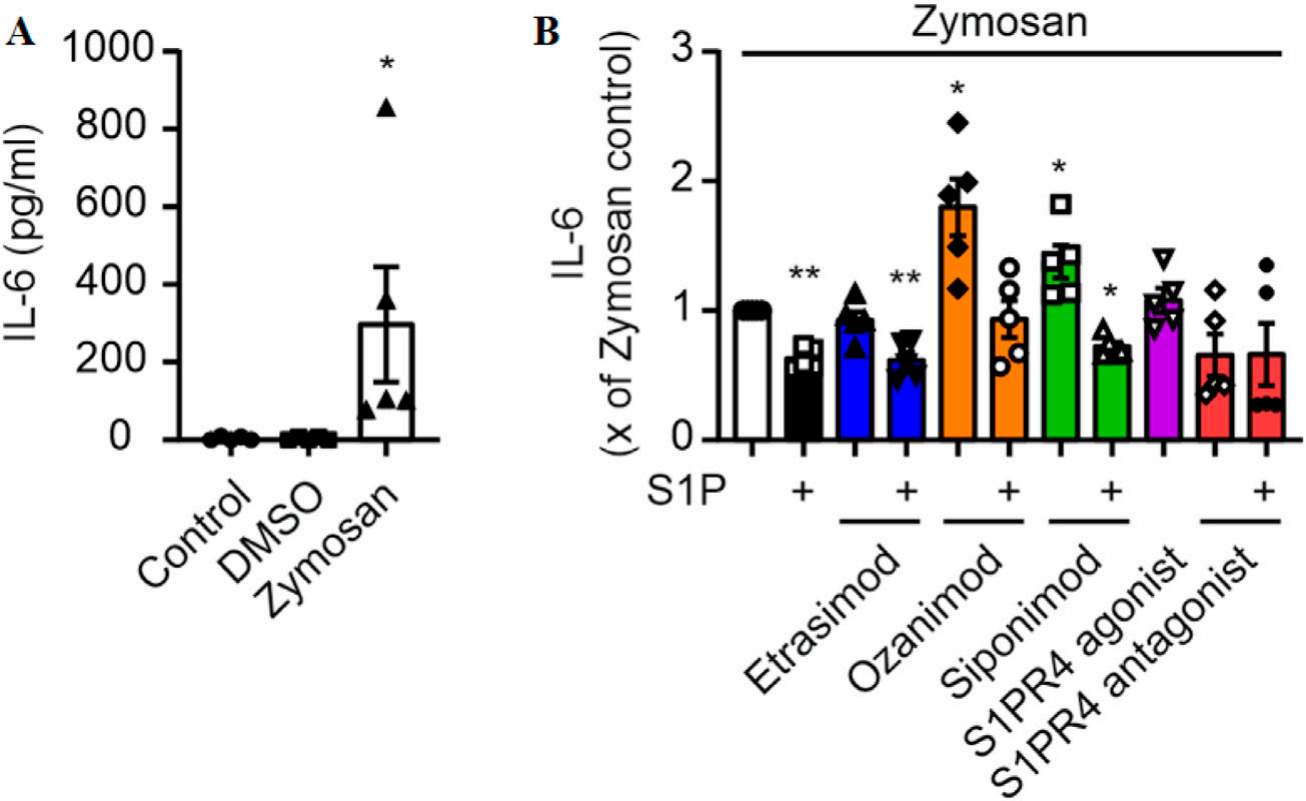
**(A)** IL-6 production by macrophages and stimulation with Zymosan A (50 μg/ml) for 24 h (data shown: mean + SEM, n = 5, comparison with one-way ANOVA, * = p < 0.05, and **(B)** the influence of S1P and S1PR modulators (100 nM each) on this production. (data shown: mean + SEM, n = 8, comparison with one sample t-test compared to Zymosan stimulation, * = p < 0.05, ** = p < 0.005).

In order to elucidate the impact of S1PR4 modulation on macrophages in the human system, we exposed human monocyte-derived macrophages to Zymosan, a TLR2 agonist capable of inducing pro-inflammatory cytokines in various immune cells (Abel and Czop, 1992; Adachi et al., 1997), and S1P with and without S1PR modulators and first determined the production of IL-6. This was based on previous data indicating a role of S1P in regulating IL-6 and chemokine production in murine macrophages in inflammation models, including peritoneal Zymosan injection (Schuster et al., 2020). While Zymosan induced production of IL-6 (Figure 1A), interestingly, S1P rather reduced Zymosan-induced IL-6 production in human macrophages (Figure 1B). The production of IL-6 was also reduced upon addition of S1P in combination with Etrasimod and Siponimod, while Ozanimod and Siponimod alone increased IL-6 production (Figure 1B). However, S1PR4 signaling had no effect on IL-6 production as neither Etrasimod alone nor the selective S1PR4 agonist CYM 50308 (Urbano et al., 2011) nor the selective S1PR4 antagonist CYM 50358 (Guerrero et al., 2011) significantly affected IL-6 production.

### 3.2 S1PR4 signaling attenuates CCL20 and CXCL5 production by macrophages

Next, we screened for chemokine production using the Legendplex Pro-Inflammatory Chemokine Panel. Out of the 13 tested chemokines, only ten were above the detection limit (see Supplementary Figure S2). Out of those ten, two chemokines were both clearly induced by Zymosan stimulation and modulated by S1PR signaling.

**FIGURE 2:**
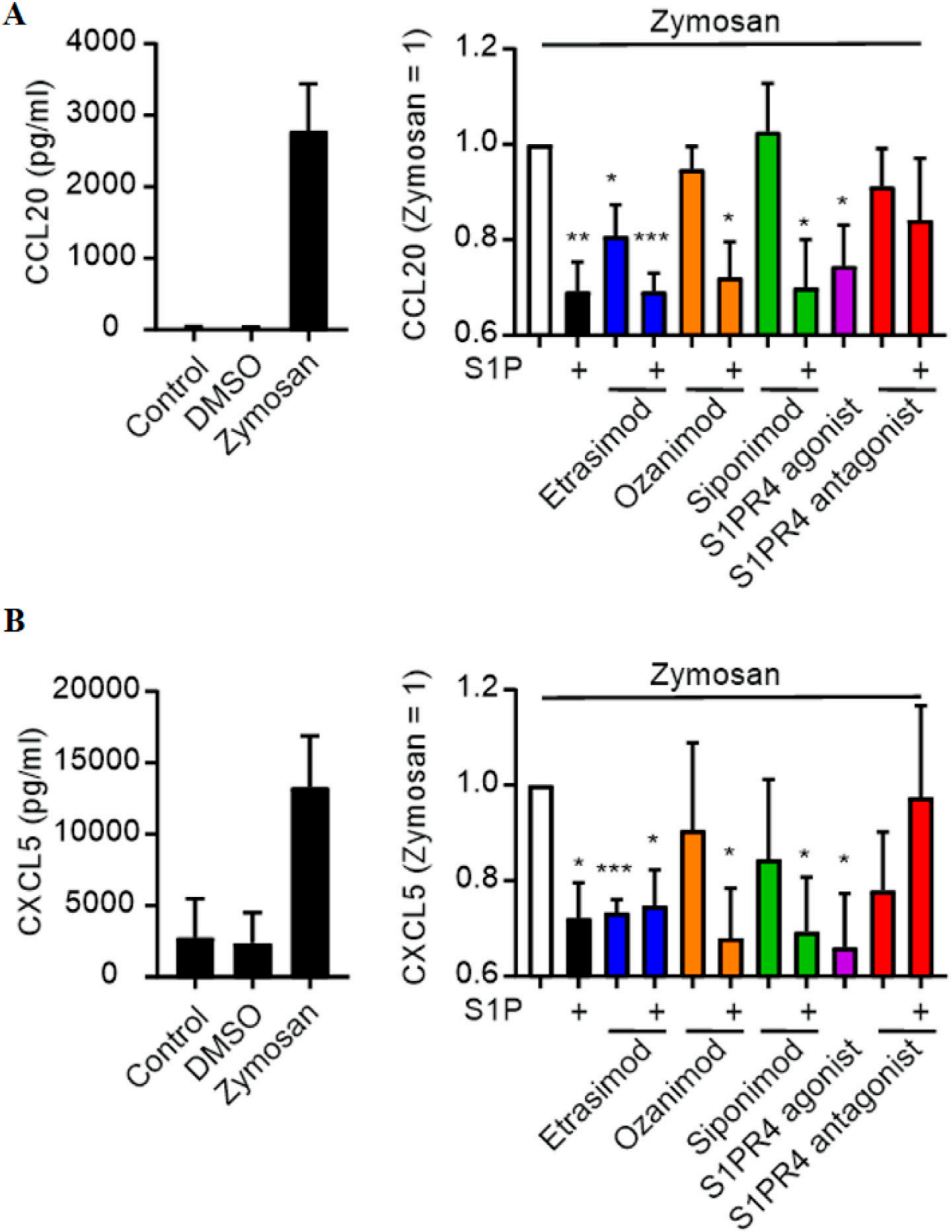
Influence of S1PR modulation (100 nM each) on **(A)** CCL20 and **(B)** CXCL5 secretion by macrophages after stimulation with Zymosan A (50 μg/ml) (data shown: mean + SEM, n = 8, comparison with one sample t-test compared to Zymosan stimulation, * = p < 0.05, ** = p < 0.005, *** = p < 0.0005).

CCL20 and CXCL5 production by Zymosan-stimulated macrophages was reduced by S1P alone and in combination with Etrasimod, Ozanimod and Siponimod (Figure 2). Notably, also Etrasimod alone and the S1PR4 agonist reduced the production of CCL20 and CXCL5, indicating that S1PR4 specifically plays a role in the regulation of those two chemokines, and that Etrasimod might work as an S1PR4 agonist.

### 3.3 Knockdown of S1PR4 in macrophages confirms its role in CCL20 and CXCL5 regulation

**FIGURE 3:**
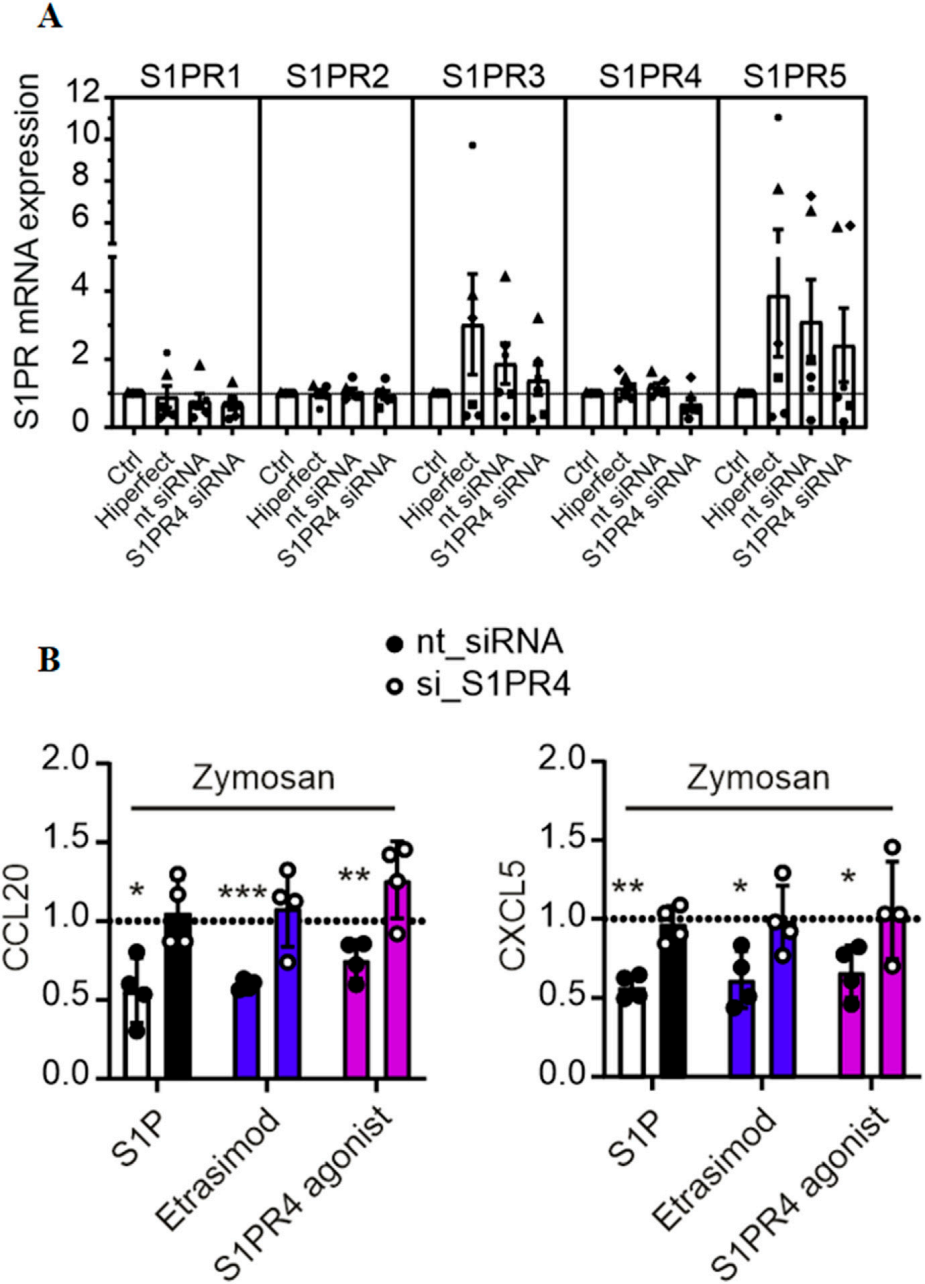
**(A)** mRNA expression levels of S1PR1-5 in macrophages after knockdown of S1PR4 with siRNA, normalized to control (data shown: mean + SEM, n = 6). **(B)** CCL20 and CXCL5 production by S1PR4-KD macrophages after stimulation with Zymosan A (50 μg/ml) and modulation with S1P, Etrasimod and the S1PR4 agonist (100 nM each), normalized to Zymosan treatment (data shown: mean + SEM, n = 4, comparison with one sample t-test compared to Zymosan stimulation, * = p < 0.05, ** = p < 0.005, *** = p < 0.0005).

In order to verify the involvement of S1PR4 in CCL20 and CXCL5 expression, we attempted to knock down S1PR4 in human macrophages prior to activation with Zymosan and S1PR modulators. S1PR4 siRNA successfully reduced S1PR4 expression in human macrophages (Figure 3A). The minor reduction in S1PR1 caused by the S1PR4 siRNA might indicate a possible regulatory interaction of these two S1P receptors. While all tested conditions showed high variations for S1PR3 and S1PR5, their absolute baseline expression levels in macrophages (see Supplementary Figure S3) were extremely low. Therefore, these variations are expected to be background noise, due to low expression levels. On top of that, macrophages were sourced from human buffy coats, which results in a certain degree of variation in basal expression levels that is expected to naturally occur between different blood donors.

The reduction of CCL20 and CXCL5 via S1P, Etrasimod and S1PR4 agonist described above was only reproduced in the control (nt siRNA) but not in the S1PR4-KD cells (Figure 3B). For all three of the treatments, the reduction of both CCL20 and CXCL5 in the nt siRNA cells was statistically significant compared to untreated cells, whereas the same treatments showed no reduction in S1PR4-KD cells. These findings confirm that S1PR4 is essential for regulating of CCL20 and CXCL5 upon Zymosan stimulation in human macrophages.

### 3.4 S1PR4 plays a role in attenuating IFN-α production by pDCs

**FIGURE 4:**
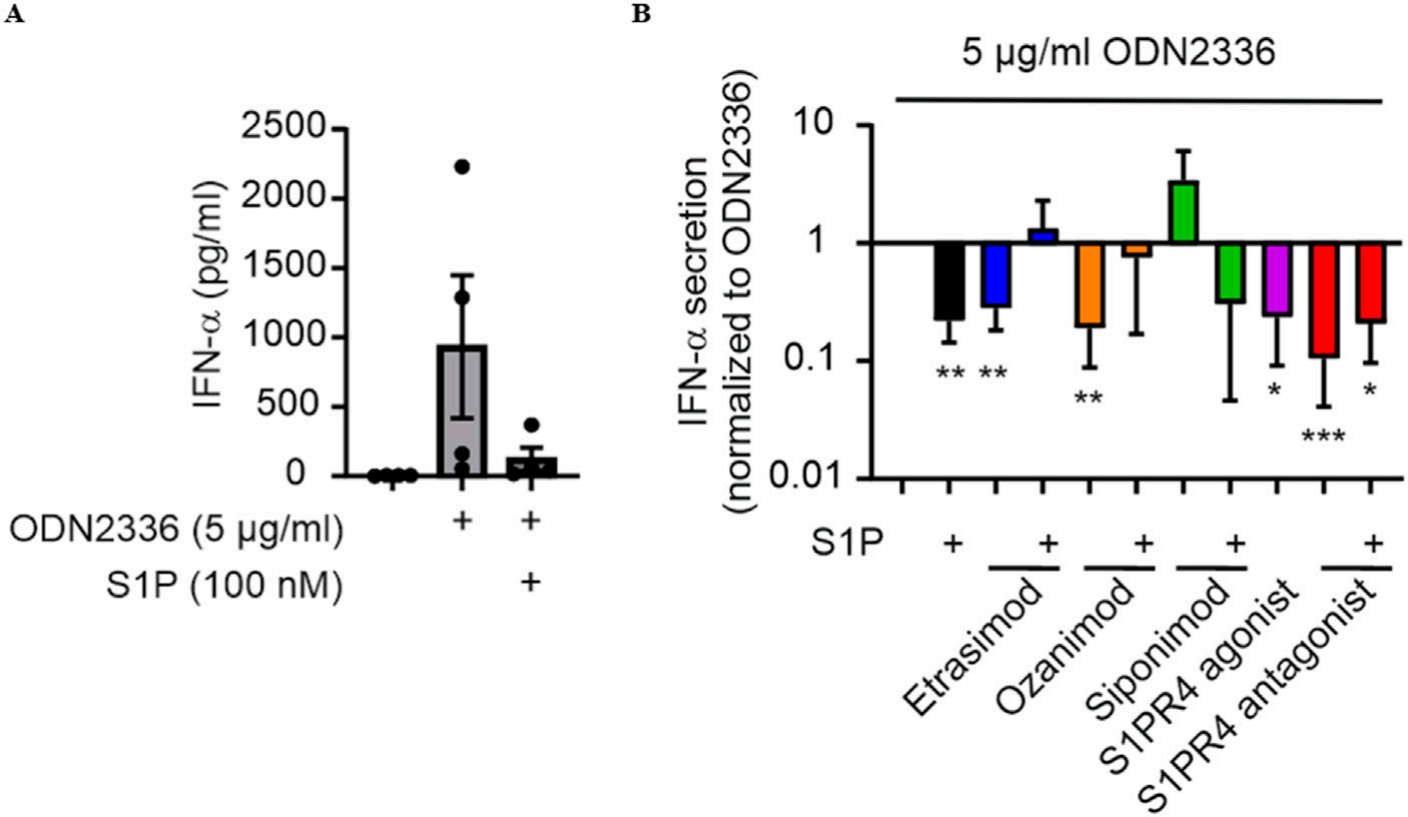
**(A)** IFN-α production by pDCs after stimulation with the TLR9 agonist ODN 2336 and S1P modulation. **(B)** Influence of S1P and S1PR modulators (100 nM each) on IFN-α production by pDCs after stimulation with ODN2336. (data shown: mean + SEM, n = 4, comparison with one sample t-test compared to ODN2336 stimulation, * = p < 0.05, ** = p < 0.005, *** = p < 0.0005).

Previous data indicated a role for S1PR4 in limiting IFN-α production by human pDCs (Dillmann et al., 2016). We next asked if Etrasimod might also affect IFN-α production. ODN 2336, a TLR9-agonistic oligonucleotide capable of stimulating pro-inflammatory cytokine release (Kumagai et al., 2008), induced IFN-α production by human pDCs (Figure 4A). IFN-α production induced by ODN was significantly decreased by addition of either S1P, Etrasimod, Ozanimod, or the S1PR4 agonist alone, while addition of S1P to Etrasimod and Ozanimod-treated pDCs abolished this effect. The S1PR4 antagonist also decreased IFN-α production induced by ODN, in this case alone and with S1P together (Figure 4B). These data indicate a more complex role of S1P receptors in regulating IFN-α production by human pDCs.

### 3.5 S1PR4 modulation increases survival of PMA-stimulated neutrophils

**FIGURE 5:**
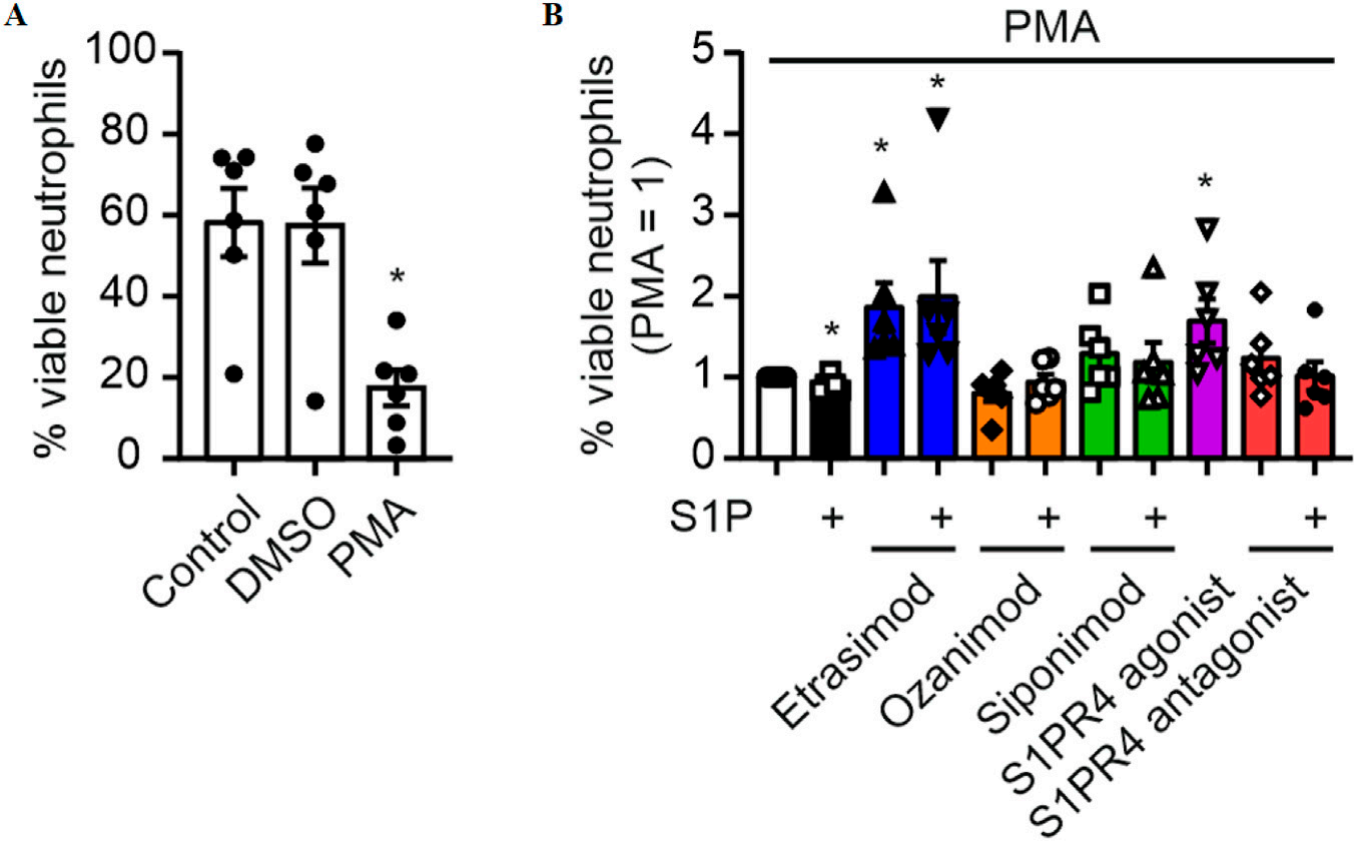
**(A)** Survival of neutrophils after stimulation with 20 nM PMA (data shown: mean + SEM, n = 6, comparison with one-way ANOVA, * = p < 0.05) and **(B)** the influence of S1PR modulation (100 nM each) on this survival (data shown: mean + SEM, n = 6, comparison with one sample t-test compared to PMA stimulation, * = p < 0.05).

To investigate a role of Etrasimod/S1PR4 in the activation of neutrophils, human Buffy Coat-derived neutrophils were stimulated with PMA, a potent activator of neutrophils via proteinkinase C signaling and subsequent autocytotoxic oxygen radical release (Takei et al., 1996), with and without S1PR modulators. As expected, after stimulation with PMA, the percentage of surviving neutrophils was substantially decreased (Figure 5A). Addition of S1P slightly increased, while Etrasimod and the S1PR4 agonist prevented neutrophil death (Figure 5B). While the effect of S1P alone was statistically significant, its magnitude (a reduction of 12% in normalized data (Figure 5B), and a total change in viable neutrophils from 20% to 18% comparing PMA alone to the combination of S1P and PMA) questions biological relevance. These findings indicate that S1PR receptors other than S1PR4 might promote neutrophil death, while S1PR4 signaling increases the survival of PMA-stimulated human neutrophils. Since neutrophil death is coupled to their activation, these data may indicate decreased activation of human neutrophils dependent on S1PR4.

### 3.6 S1PR4 modulation reduces neutrophil activation as measured by activated CD11b

**FIGURE 6:**
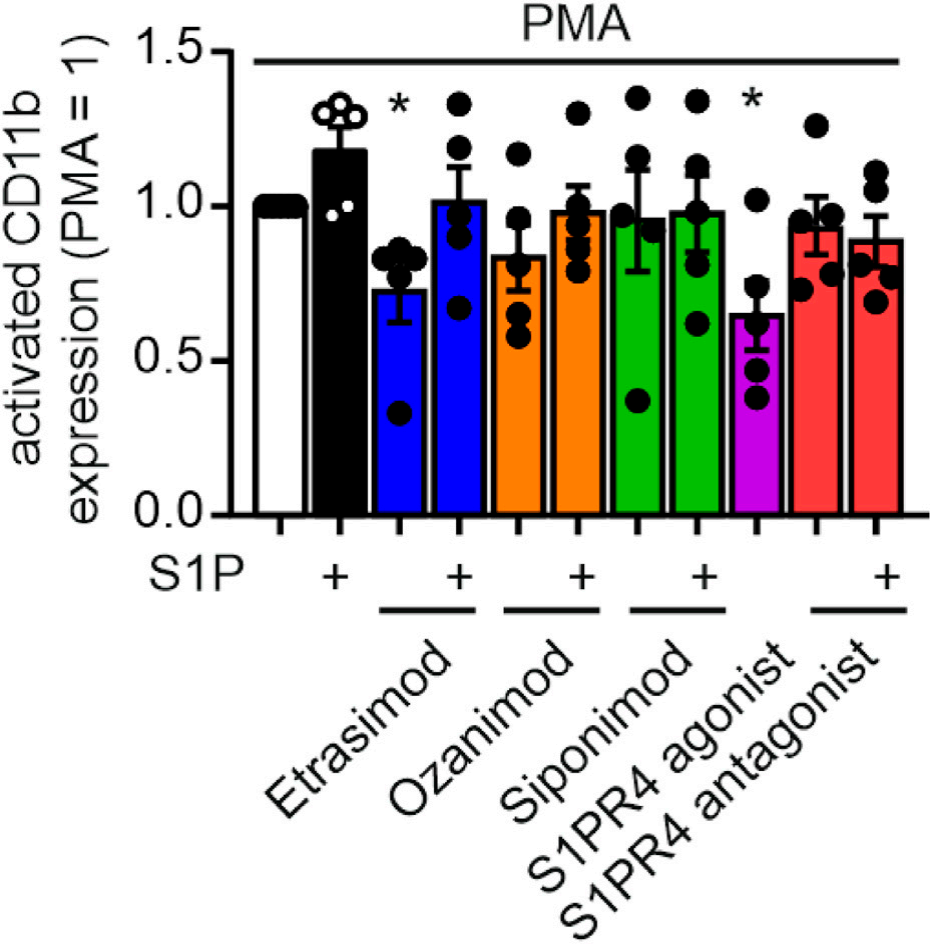
Activation of neutrophils measured by activation epitope of CD11b after PMA stimulation (20 nM) and S1PR modulation (100 nM each) (data shown: mean + SEM, n = 5, comparison with one sample t-test compared to PMA stimulation, * = p < 0.05).

To analyze the impact of Etrasimod on neutrophil activation, exposure of an CD11b epitope that indicates activation of this integrin was analyzed. Both Etrasimod and the S1PR4 agonist reduce the expression of the activated CD11b epitope (Figure 6), while overall CD11b expression was not affected (see Supplementary Figure S4). Thus, the activation of neutrophils was reduced by Etrasimod, which confirms the association of increased survival correlating with decreased activation.

### 3.7 Etrasimod and Ozanimod internalize S1PR1 and S1PR4 upon stimulation

To analyze how S1PR4 dynamics are affected by Etrasimod, we used S1PR4-specific antibodies in flow cytometry and Western Blotting. Antibodies targeting S1PR4 that we employed in the past were generally of low quality. Therefore, in order to validate further Western Blotting antibodies and to analyze internalization dynamics, we used a CHO-K1 cell line transfected with either human S1PR1 (CHO_S1PR1) or human S1PR4 (CHO_S1PR4). These cell lines express only S1PR1 or S1PR4, respectively, but not the other S1PR (see Supplementary Figure S5).

In the cyclic process of its regulation, S1PR4 is sequestered from the cell surface upon agonist stimulation. Due to this internalization, further S1PR4 signaling is prevented until the agonist concentration falls beneath a certain threshold, and the cycle can begin anew (Gräler et al., 2003). This means that S1PR4 surface expression can serve as a readout for receptor activation.

**FIGURE 7:**
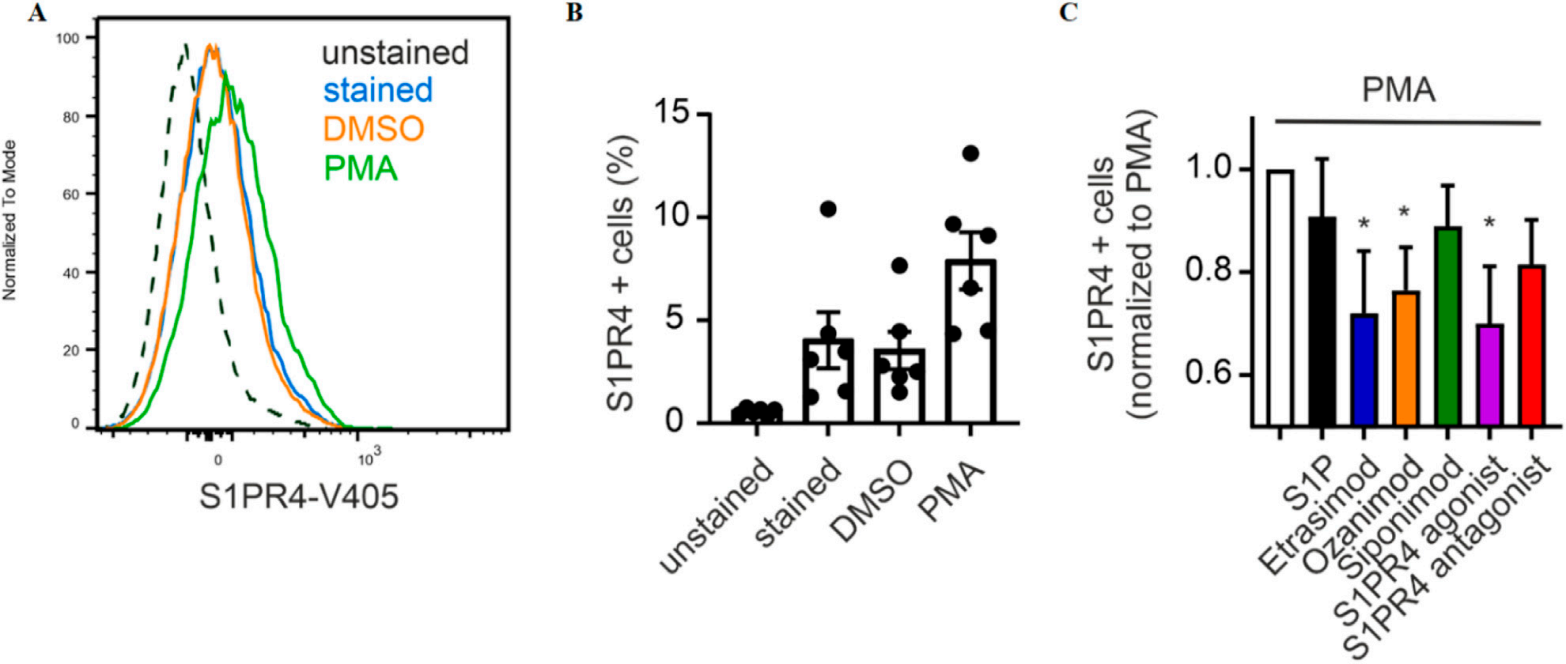
**(A)** Exemplary signal intensity of S1PR4 expression, **(B)** percentage of neutrophils expressing S1PR4 and **(C)** influence of S1P signaling on S1PR4 expression (data shown: mean + SEM, n = 6, comparison with one sample t-test compared to PMA stimulation, * = p < 0.05).

First, we employed an S1PR4 flow cytometry antibody for analyzing its surface expression on primary human neutrophils after S1PR modulation. For that, we established that the antibody we used showed a distinct signal intensity in stained over unstained cells, and this signal intensity was increased by activation with PMA (Figure 7A). The percentage of S1PR4^+^ cells increased markedly upon PMA stimulation compared to untreated control and vehicle (Figure 7B). Moreover, addition of Etrasimod, Ozanimod and the S1PR4 agonist CYM 50308 reduced the surface expression of S1PR4 induced by PMA (Figure 7C). While the same trend was visible for S1P, the extent was not as pronounced as for the S1PR modulators. This implicates S1P itself as not as strong an S1PR4 agonist as Etrasimod or CYM 50308 in our cellular system. While a recent analysis of S1PR dynamics (Gaidarov et al., 2024) showed S1P as the stronger S1PR4 agonist, they employed transfected cell lines while our data here is obtained from primary cells. Additionally, we measured S1PR4 surface expression as a more functional readout, while Gaidarov et al. focused on the more mechanistical approach of a β-arrestin assay. A direct comparison between results obtained by different methods might be challenging, and a more in-depth study of howthese compounds differentially affect receptor dynamics (such as recycling to the plasma membrane) would be needed for the full context. Ozanimod, however, has only recently been shown to also act on S1PR4 (Gaidarov et al., 2024), and we also noted a decreased surface expression of S1PR4 after Ozanimod treatment. This might also indicate co-signaling or interaction between S1PR4 and one or more of the other S1PRs, and indeed, cell surface association of S1PR1 and S1PR4 has been observed before (Matsuyuki et al., 2006).

**FIGURE 8:**
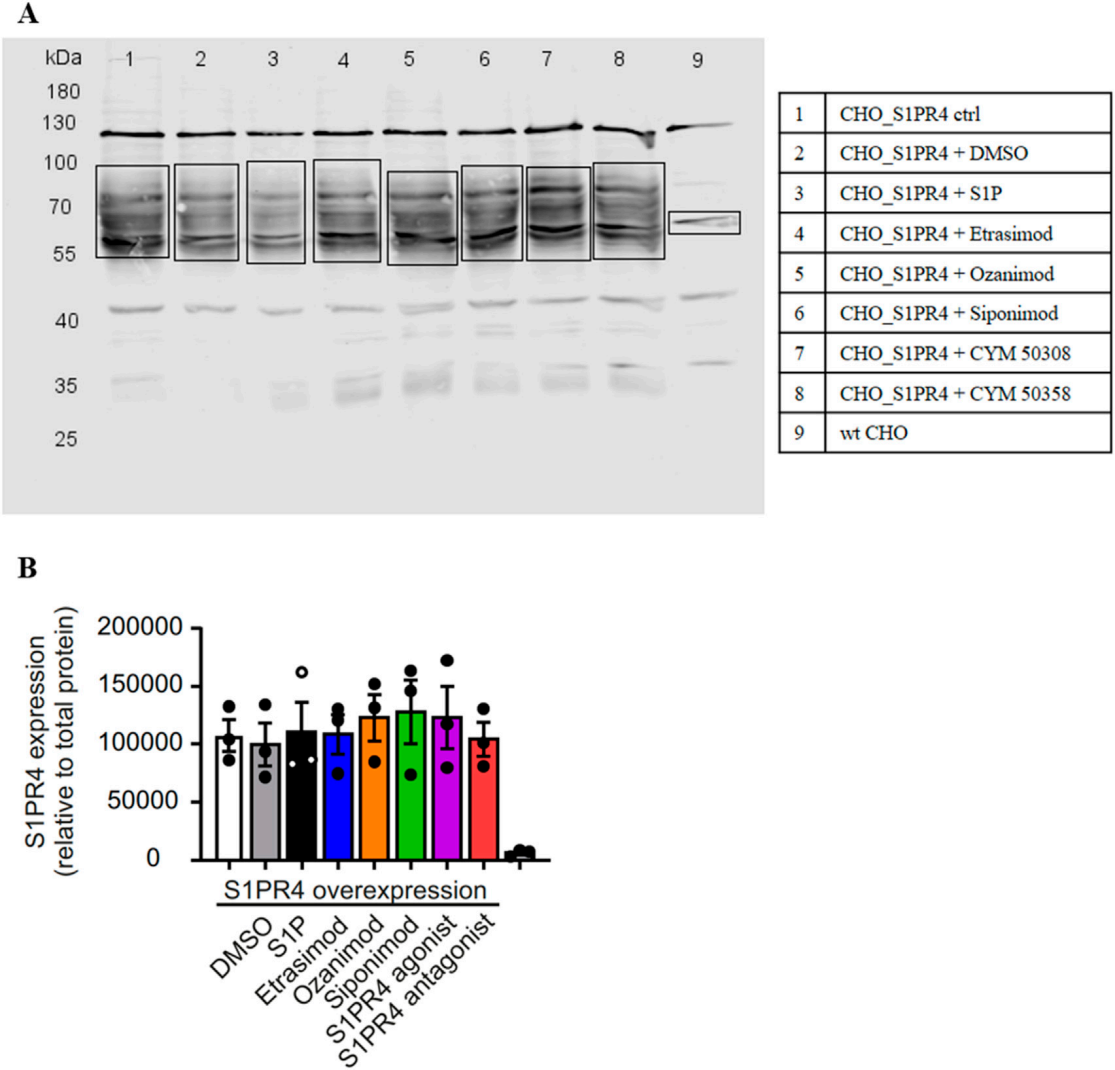
Western Blot of CHO_S1PR4 cell line with S1PR4 antibody. **(A)** Representative Western Blot. **(B)** Quantification of S1PR4 expression relative to total protein staining.

Global S1PR4 expression was clearly increased in the CHO_S1PR4 cells compared to wt CHO cells (Figure 8A). Importantly, short-time treatment (30 min) with S1PR modulators did not affect global S1PR4 expression (Figure 8B). This indicates that while S1PR4 is reduced on the cell surface, it seems to be internalized but not degraded, since the overall amount stays the same.

**FIGURE 9:**
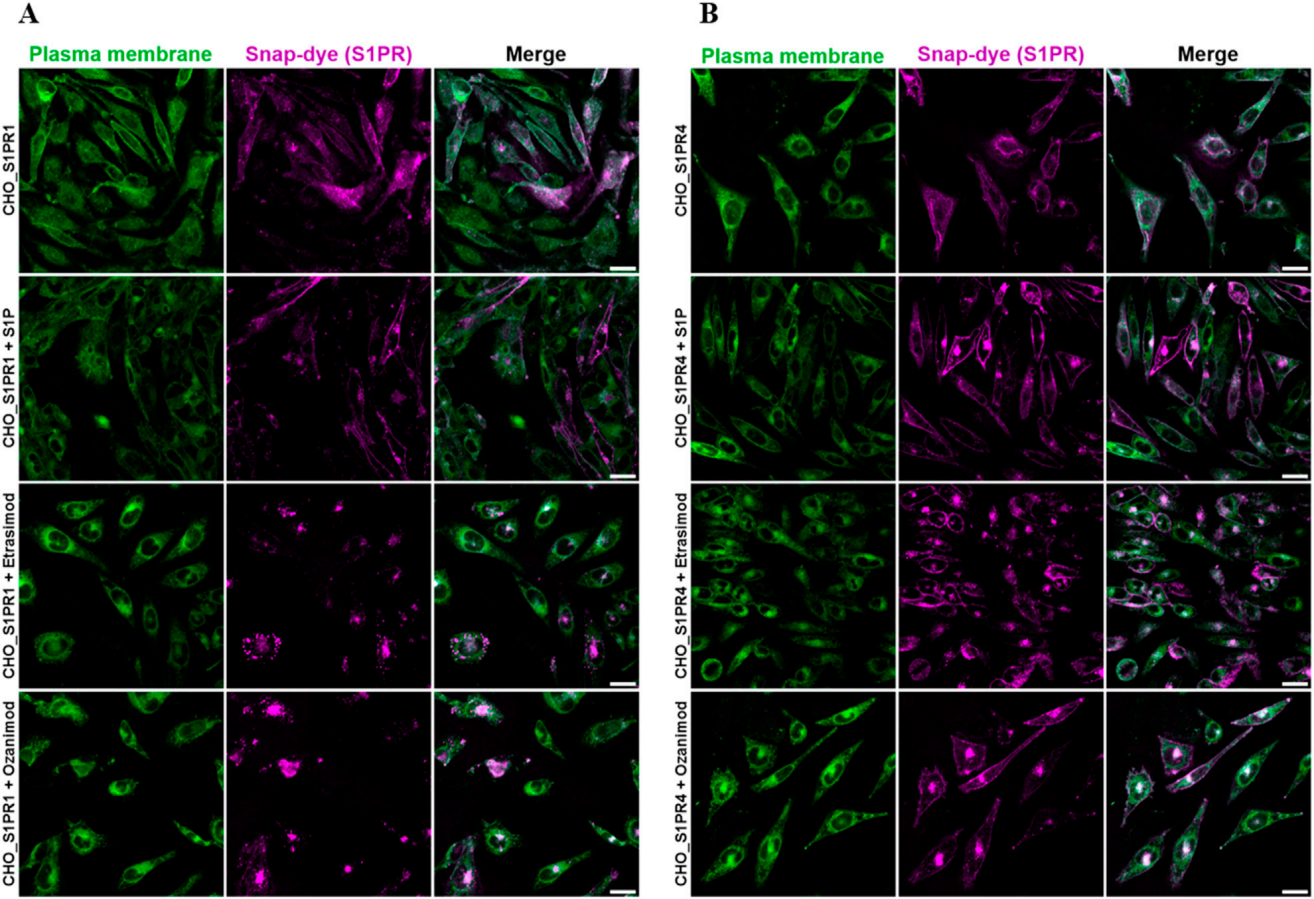
Representative images of confocal microscopy of **(A)** CHO_S1PR1 and **(B)** CHO_S1PR4 cells either untreated or treated with 100 nM S1P, 100 nM Etrasimod or 100 nM Ozanimod for 60 min. Plasma membrane staining is shown in the first column, SNAP-tag dye in the second, and the composite of both channels in the third, respectively. Scale bar marks 20 μm.

To gain a deeper understanding of S1PR dynamics, we employed confocal microscopy of S1PR cell lines as an alternative strategy to analyze their internalization. Briefly, a CHO cell line expressing SNAP-tagged S1PR1 or S1PR4, respectively, was treated with S1P, Etrasimod or Ozanimod. The SNAP-dye is only present in the transfected but not the parent cell line (see Supplementary Figure S6), and treatment with S1P, Etrasimod and Ozanimod showed bright concentrated spots of internalized receptors for both cell lines (Figure 9).

In addition to this visual qualification of S1P receptor dynamics, we next quantified the localization of the SNAP-tagged receptor in both the plasma membrane and the cytoplasm, and then calculated the ratio between the two compartment-localized fluorescence signals to obtain a deeper insight into the receptor dynamics upon stimulation.

**FIGURE 10:**
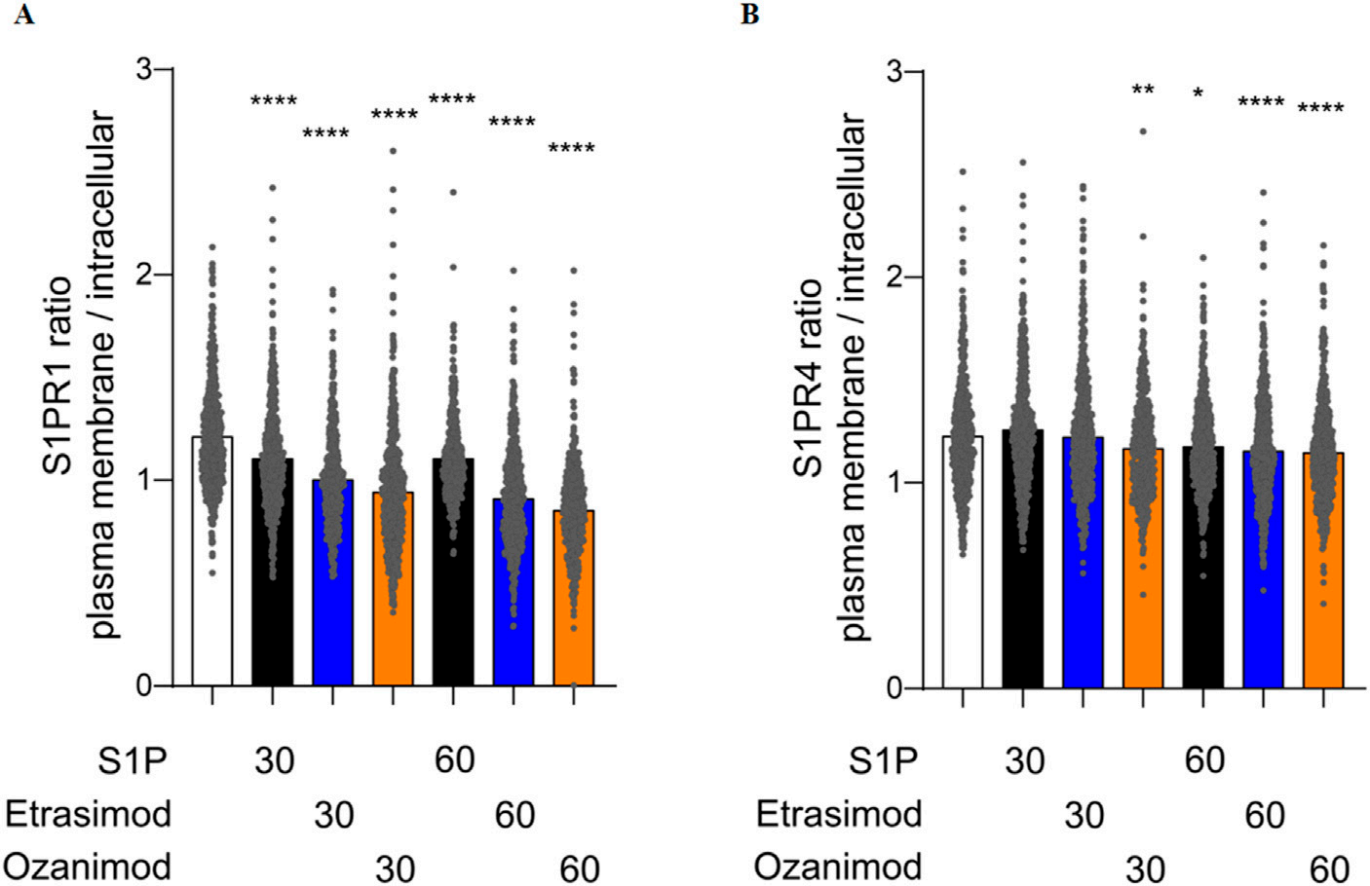
Quantification of the ratio between plasma membrane and intracellular localization of **(A)** S1PR1 and **(B)** S1PR4 after treatment with 100 nM S1P, Etrasimod or Ozanimod for 30 or 60 min (data shown: mean, Kruskal-Wallis test compared to control, * = p < 0.05, ** = p < 0.005, *** = p < 0.0005, **** = p < 0.0001).

S1P, Etrasimod and Ozanimod internalized the S1PR1 compared to untreated cells, both after 30 and 60 min (Figure 10A). The effect was more pronounced with Etrasimod than with S1P, and even more pronounced with Ozanimod, which confirms that both modulators are potent S1PR1 agonists. For the S1PR4 cell line, the only internalization noticeable after 30 min was with Ozanimod. However, after 60 min, also S1P and Etrasimod showed receptor internalization (Figure 10B). The internalization of S1PR4 was overall less pronounced than that of S1PR1 upon the same stimuli, which might indicate either a lower level of agonism or a different time course for S1PR4 activation.

## 4 Discussion

With the work presented above, we were able to show that IL-6 production by Zymosan-stimulated macrophages was reduced by S1P and increased by Ozanimod and Siponimod. However, there was no effect of S1PR4 signaling on IL-6 production as neither Etrasimod alone nor the S1PR4 agonist/antagonist significantly affected IL-6 production. The fact that Ozanimod and Siponimod increased IL-6 levels, while S1P addition still was able to reduce them, indicates an involvement of S1PR2/3 in regulating IL-6 levels upon stimulation of human macrophages with Zymosan. Previous studies in murine macrophages did find an involvement of both S1PR2 (Yu, 2016) and S1PR3 (Heo and Im, 2019) in the regulation of IL-6 production, which corroborates our observation. However, in contrast to Ozanimod and Siponimod, Etrasimod did not elevate IL-6 levels, which may be favorable under inflammatory disease conditions, since IL-6 signaling and subsequent T cell activation have been implicated in IBD pathogenesis (Atreya et al., 2000; Yamamoto et al., 2000), and therefore not further increasing IL-6 levels would be preferable.

Etrasimod and S1PR4 were shown to be involved in the downregulation of CCL20 and CXCL5 production in Zymosan-stimulated macrophages, which indicates that Etrasimod works as an S1PR4 agonist. Importantly, CCL20 is known to recruit pathogenic lymphocytes by acting on its receptor CCR6 in colitis and molecules targeting the CCL20/CCR6 axis are already in clinical development (Allodi et al., 2023). Moreover, CXCL5 was recently reported to recruit neutrophils towards sites of ulceration in UC (Friedrich et al., 2021), making it a valid novel drug target (Chen et al., 2023). These data suggest that S1PR4 signaling in macrophages might be beneficial for limiting inflammation in colitis.

Surprisingly, our studies showed that not only Etrasimod, but also Ozanimod was able to attenuate IFN-α production by ODN2336-stimulated pDCs in an S1PR4-speciffic manner. Although it might be tempting to incriminate S1PR1 and S1PR5 signaling in this activity of Ozanimod, our own data on S1PR4 internalization, and also some recently published results on pharmacological properties of S1PR modulators (Gaidarov et al., 2024) support the notion that Ozanimod might also signal through S1PR4, which would explain the similar behavior of Etrasimod and Ozanimod in the regulation of IFN-α production. The inhibitory effect of S1P on IFN-α production has previously been attributed to both S1PR4 and S1PR1 (Dillmann et al., 2016; Teijaro et al., 2016). However, the finding that Etrasimod by itself strongly reduced IFN-α production, while its pre-incubation fully blocked the inhibitory effect of S1P on IFN-α production, is consistent with a role of Etrasimod as an S1PR4 superagonist/functional antagonist. While the S1PR4 antagonist CYM 50358 shows very high selectivity for S1PR4 over the other S1PRs (Guerrero et al., 2011), the possibility remains of other signaling pathways contributing to the ambiguous effect observed.

Additionally, neutrophil death was decreased after treatment with Etrasimod compared to PMA-stimulation alone, indicating a similar decrease in neutrophil activation. These data further support the hypothesized role of Etrasimod as a S1PR4 superagonist/functional antagonist. The supposed link between increased survival and decreased activation was investigated via expression analysis of activated CD11b, and the association was confirmed. Since activated neutrophils have been implicated in IBD severity (Söderman and Almer, 2024), reducing their activation level is promising for the improvement of disease progression. This further strengthened the notion of Etrasimod working as a S1PR4 superagonist/functional antagonist.

Lastly, we were able to show that Etrasimod, Ozanimod and the S1PR4 agonist CYM 50308 led to decreased surface expression of S1PR4 on activated neutrophils. This corroborates the expected S1PR4 internalization upon activation (Gräler et al., 2003; Oo et al., 2007). Analyzing receptor internalization via confocal microscopy confirmed agonistic activity of S1P, Etrasimod and Ozanimod on S1PR4, although to a lesser extent than on S1PR1. These findings are supported by recently published data that S1P, Etrasimod and Ozanimod can induce β-arrestin signaling via S1PR4, which leads to subsequent receptor internalization (Gaidarov et al., 2024). However, that same study showed that only S1P but not Etrasimod or Ozanimod induce G-protein dependent signaling of S1PR4. Based on this information, it appears possible that regulation of CCL20 and CXCL5 production by macrophages as well as neutrophil activation via PMA may depend on S1PR4 β-arrestin signaling, while the more complex regulation of IFN-α production by pDCs might be either G-protein dependent or controlled by another receptor entirely. Thus, further research in the exact mechanics of S1PR4 activation is required.

Taken together, all these observations indicate that Etrasimod might work as a superagonist/functional antagonist of the S1PR4 receptor, and further strengthen an essential role of S1PR4 signaling in an pro-inflammatory immune context such as in IBD.

## Data Availability

The raw data supporting the conclusions of this article will be made available by the authors, without undue reservation.
