# Supplementary material for: S1PR4-dependent effects of Etrasimod on primary human myeloid immune cell activation
Source: Front Pharmacol. 2025 Apr 24;16:1590816. doi: 10.3389/fphar.2025.1590816 (PMC12058506; doi:10.3389/fphar.2025.1590816)
Supplement: Supplementary file 1 [file DataSheet1.pdf]

Supplementary Materials

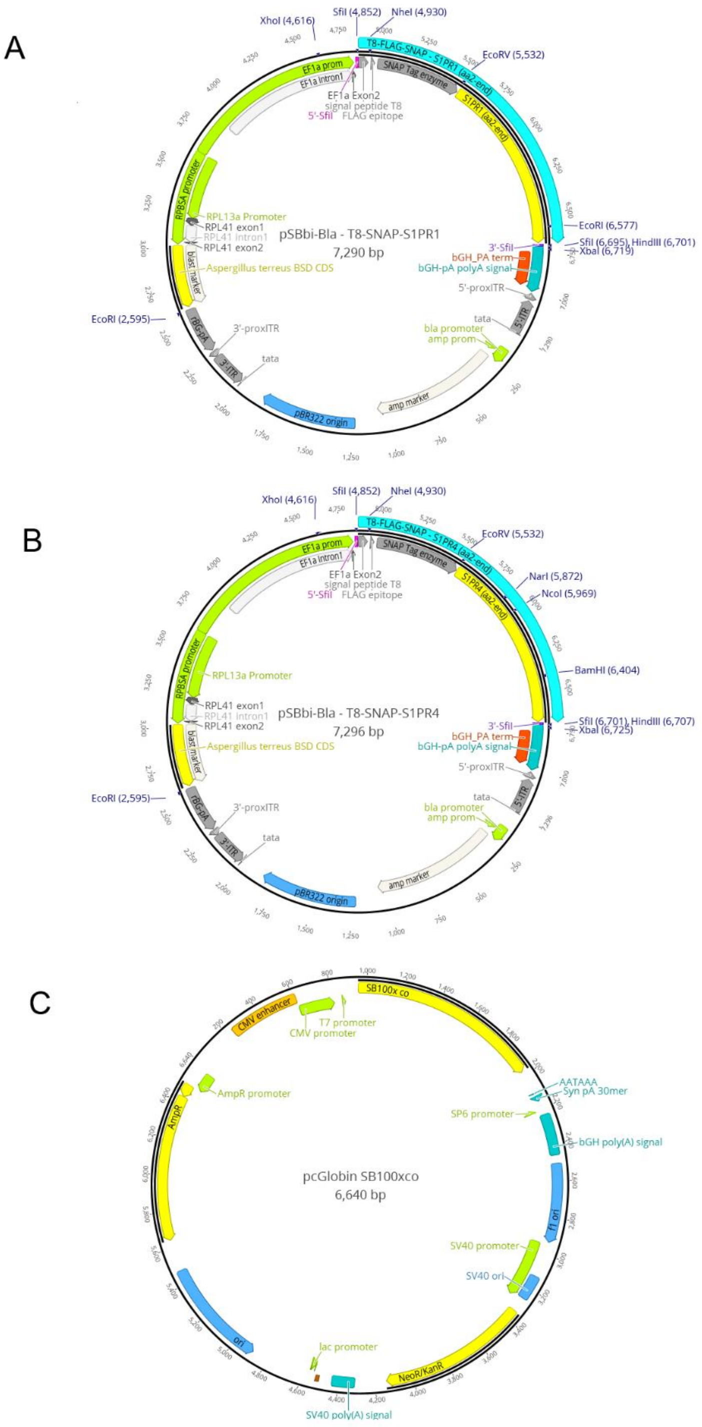

Supplementary Figure 1: Plasmid maps for **(A)** the S1PR1, **(B)** the S1PR4 and **(C)** the sleeping beauty transposase plasmids used for the generation of the CHO_S1PR1 and CHO_S1PR4 cell lines.

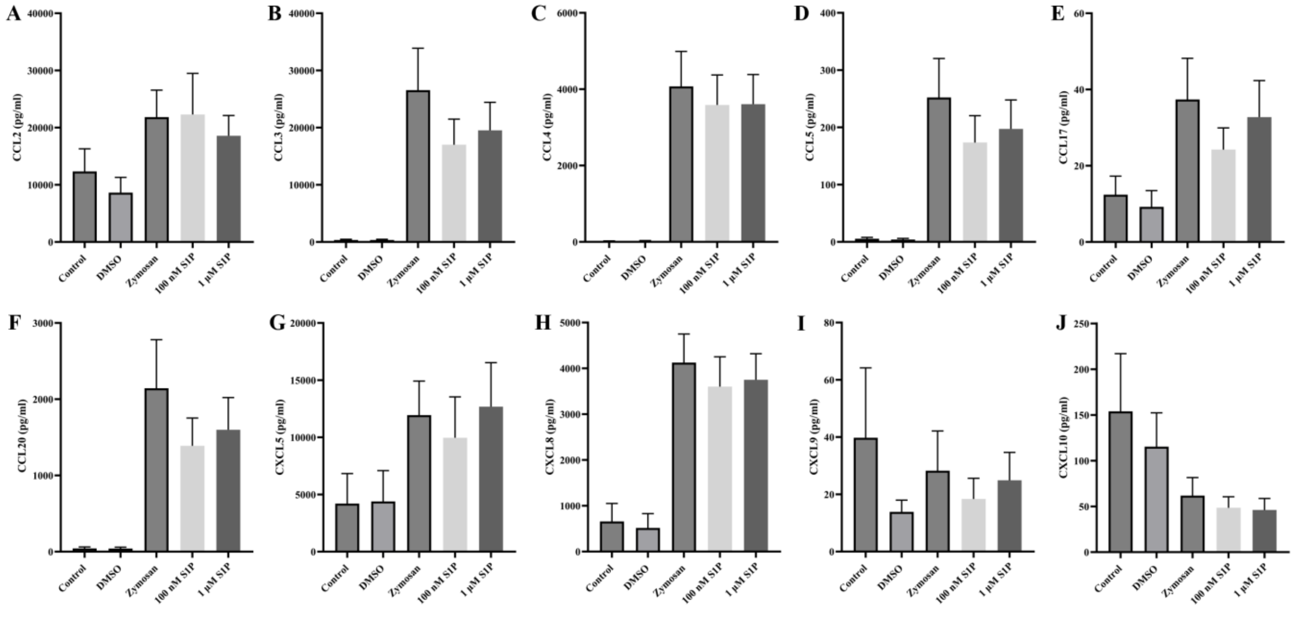

Supplementary Figure 2: (A)-(J) Chemokine secretion by macrophages after addition of S1P (100 nM or 1 μM) and stimulation with Zymosan A (50 μg/ml) for 24 h (data shown: mean + SEM, n = 8).

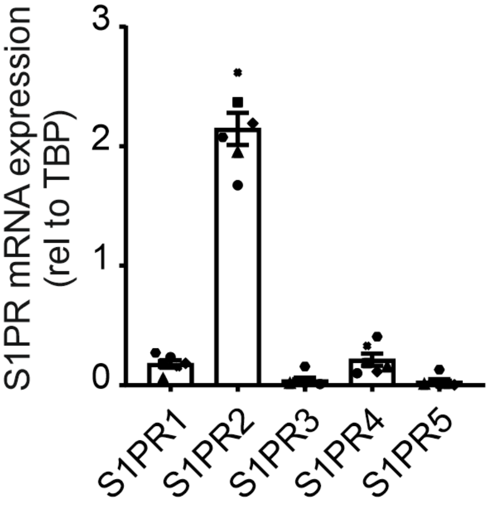

Supplementary Figure 3: mRNA expression levels of S1PR1-5 in macrophages (data shown: mean + SEM, n = 6).

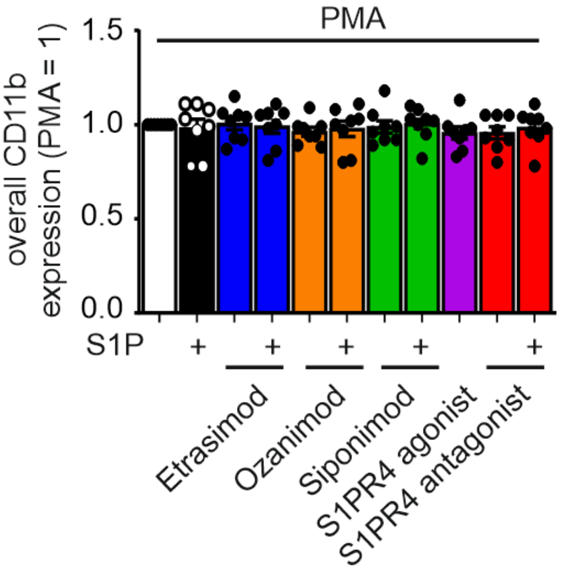

Supplementary Figure 4: Expression level of overall CD11b in PMA-stimulated neutrophils (data shown: mean + SEM, n = 8).

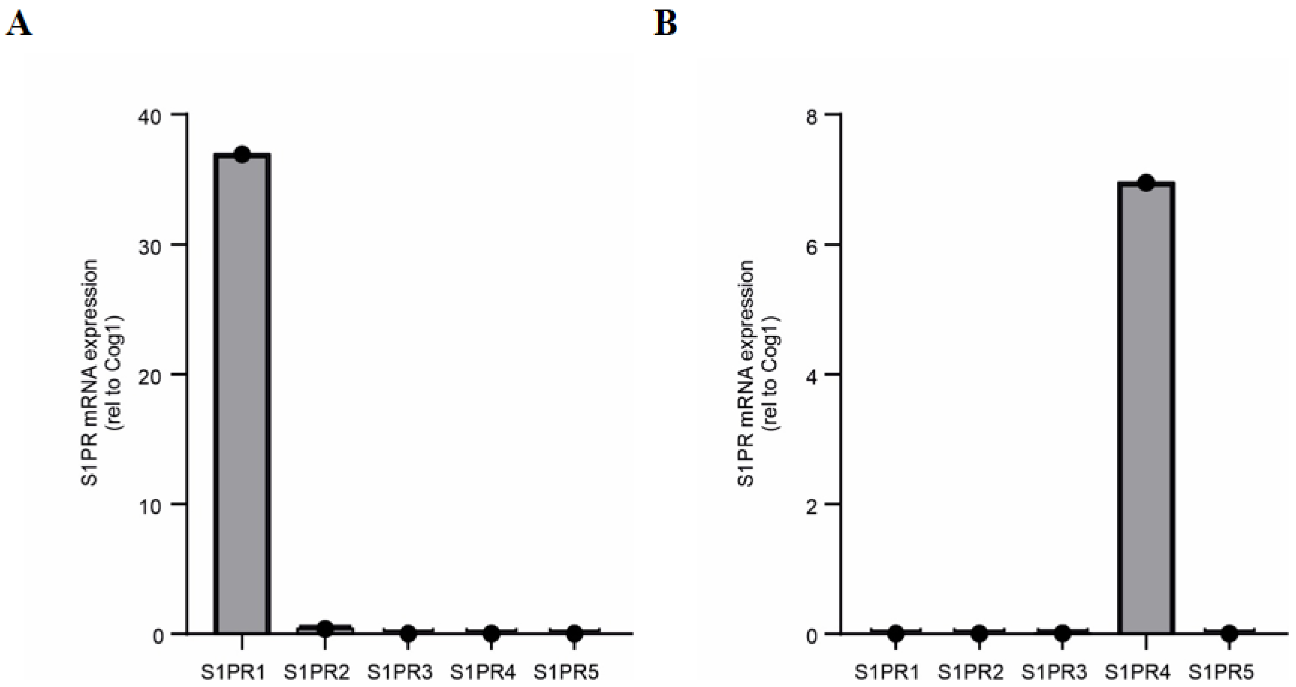

Supplementary Figure 5: Expression level of human S1PR in **(A)** the CHO_S1PR1 and **(B)** the CHO_S1PR4 cell lines.

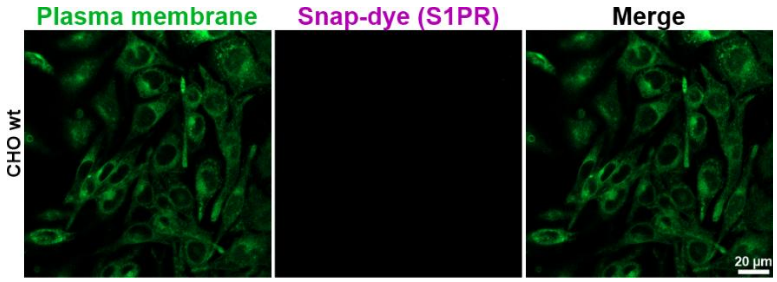

Supplementary Figure 6: Representative image of confocal microscopy of CHO-K1 wt cells. Plasma membrane staining is shown in the first column, SNAP-tag dye in the second, and the composite of both channels in the third.
